# The impact of the SARS-CoV-2 pandemic on the prevalence of respiratory tract pathogens in patients with community-acquired pneumonia in Germany

**DOI:** 10.1080/22221751.2021.1957402

**Published:** 2021-08-01

**Authors:** Theo Dähne, Wolfgang Bauer, Andreas Essig, Bernhard Schaaf, Christoph D. Spinner, Mathias W. Pletz, Gernot Rohde, Jan Rupp, Martin Witzenrath, Marcus Panning, Members of the CAPNETZ study group

**Affiliations:** a Institute of Virology, Medical Center - University of Freiburg, Faculty of Medicine, University of Freiburg, Freiburg, Germany; b Department of Emergency Medicine, Charité-Universitätsmedizin Berlin, Freie Universität Berlin, Humboldt-Universität zu Berlin, and Berlin Institute of Health, Berlin, Germany; c Institute of Medical Microbiology and Hygiene, University Hospital of Ulm, Ulm, Germany; d Hospital Dortmund gGmbH, Dortmund, Germany; e Department of Internal Medicine II, University Hospital rechts der Isar, School of Medicine, Technical University Munich, Munich, Germany; f Institute of Infectious Diseases and Infection Control, Jena University Hospital / Friedrich-Schiller-University Jena, Jena, Germany; g CAPNETZ Stiftung, Hannover, Germany; h Department of Respiratory Medicine, Medical Clinic I, Goethe University Hospital, Frankfurt/Main, Germany; i Biomedical Research in Endstage and Obstructive Lung Disease Hannover (BREATH), German Center for Lung Research (DZL), Hannover, Germany; j Department of Infectious Diseases and Microbiology, University Hospital Schleswig-Holstein, Lübeck, Germany; k Department of Infectious Diseases and Pulmonary Medicine and the Division of Pulmonary Inflammation, Charité - Universitätsmedizin Berlin, Freie Universität Berlin, Humboldt-Universität zu Berlin, and Berlin Institute of Health, Berlin, Germany; l Associate Member of the DZL

**Keywords:** SARS-CoV-2, community-acquired pneumonia, viral pathogens, molecular methods, prospective study

## Abstract

We show a shift in the prevalence of respiratory viral pathogens in community-acquired pneumonia patients during the COVID-19 pandemic. Our data support the efficiency of non-pharmaceutical interventions on virus circulation except for rhinoviruses. The consequences of an altered circulation on subsequent winter seasons remain unclear and support the importance of systematic virological surveillance.

Community-acquired pneumonia (CAP) is a major cause of morbidity and mortality in adults worldwide. Microbiological causes of CAP comprise mainly bacteria and viruses [1]. Studies suggest that viral pathogens account for up to 35% of infections in CAP patients [2]. Recently, severe acute respiratory syndrome coronavirus 2 (SARS-CoV-2) emerged as a novel cause of CAP. Various non-pharmaceutical interventions (NPI), including social distancing and mask wearing, were initiated globally to contain the current Coronavirus disease 2019 (COVID-19) pandemic and to limit its impact on Public Health [3]. Interestingly, recent surveillance data from Australia indicated a possible effect of NPI on the circulation of seasonal influenza and the prevalence of other viral respiratory tract pathogens [4].

We used the prospective, multinational, multicentre cohort of the German study Group CAPNETZ (competence network for CAP) to comprehensively analyse the frequencies of 21 respiratory pathogens spanning three consecutive years. We exclusively used molecular methods for the detection of the most common viral pathogens and selected atypical bacteria. This included testing for adenovirus, bocavirus, coronavirus (CoV) OC43, CoV 229E, CoV HKU1, CoV NL63, enterovirus, influenza virus A + B, human metapneumonvirus (HMPV), parainfluenza virus 1-4, human parechovirus, respiratory syncytial virus A + B (RSV), rhinovirus and atypical bacteria (*Bordetella pertussis, Legionella pneumophila, Mycoplasma pneumoniae*). We collected nasopharyngeal swab samples from the upper respiratory tract between 1st January 2018 and 31st December 2020. Briefly, inclusion criteria were age ≥ 18 years, a new lung infiltrate on chest radiograph, and at least one of the following clinical findings: cough, purulent (off-white, yellow or green and opaque) sputum, fever (≥38.3 °C), or focal chest sign on auscultation. Exclusion criteria were hospitalization during 28 days preceding the study, severe immunosuppression and active tuberculosis. Each patient contributed one swab sample. Specimens were shipped frozen from each local study centre to the central testing facility in Freiburg. We processed samples immediately upon receipt using a multiplex real-time RT–PCR panel and, beginning 1st January 2020, individual SARS-CoV-2 RT–PCR. We used a commercial multiplex PCR panel: Fast Track Diagnostics respiratory pathogens 21 (Siemens Healthineers, Eschborn, Germany) as described [5]. Testing for SARS-CoV-2 was performed using the RealStar SARS-CoV-2 RT–PCR (Altona Diagnostics, Hamburg, Germany) as recommended by the manufacturer. The study was carried out following definitions of Good Clinical Practice, according to the declaration of Helsinki. We obtained signed informed consent for prospective bio banking from every single individual. Institutional Review Board (IRB) of each participating clinical centre was obtained. A central IRB approval is available by the Ethics Committee of the Hannover Medical School; project approval number: 301-2008.

A total of 1076 patients were included (n=280 in 2018, n=361 in 2019, n=435 in 2020). Median age was 66 years (range 18-98) and 370 (34%) were females (information missing for 26 patients). Rate of females was 35% in 2018, 33% in 2019, and 35% in 2020, respectively.

Overall, 313/1076 (29.1%) patients tested positive for a respiratory pathogen using molecular methods. In detail, 285/313 (91%) were positive for a viral pathogen and in 28/313 (9%) an atypical bacterial pathogen (*Mycoplasma pneumoniae* only) was detected. Co-detections were seen in 13 patients (12 with viral-viral co-detection and one with viral-bacterial co-detection).

In 2018, the most frequently detected pathogen was rhinovirus (17/280, 6%), followed by influenza virus (13/280, 4.6%), and *Mycoplasma pneumoniae* (9/280, 3.2%) (Figure 1, panel A). Interestingly, this pattern did not change in 2019, with rhinovirus (36/361, 9.9%), influenza virus (17/361, 4.7%), and *Mycoplasma pneumoniae* (14/361, 3.9%) being the most frequently detected pathogens (Figure 1, panel B). In 2020, however, the most common pathogen was SARS-CoV-2 (96/435, 22%), followed by rhinovirus (24/435, 5.5%), and influenza virus (9/435, 2%). Of note, NPI including social distancing and the recommendation to wear facemasks in public were initiated in Germany in early April 2020 [6]. Hereafter, no other viral pathogens were detected in our cohort except for SARS-CoV-2 and rhinovirus (Figure 1, panel C). Interestingly, 7/96 (7.3%) SARS-CoV-2 positive samples did show co-detections and all were associated with the detection of rhinovirus.

**Figure 1.**
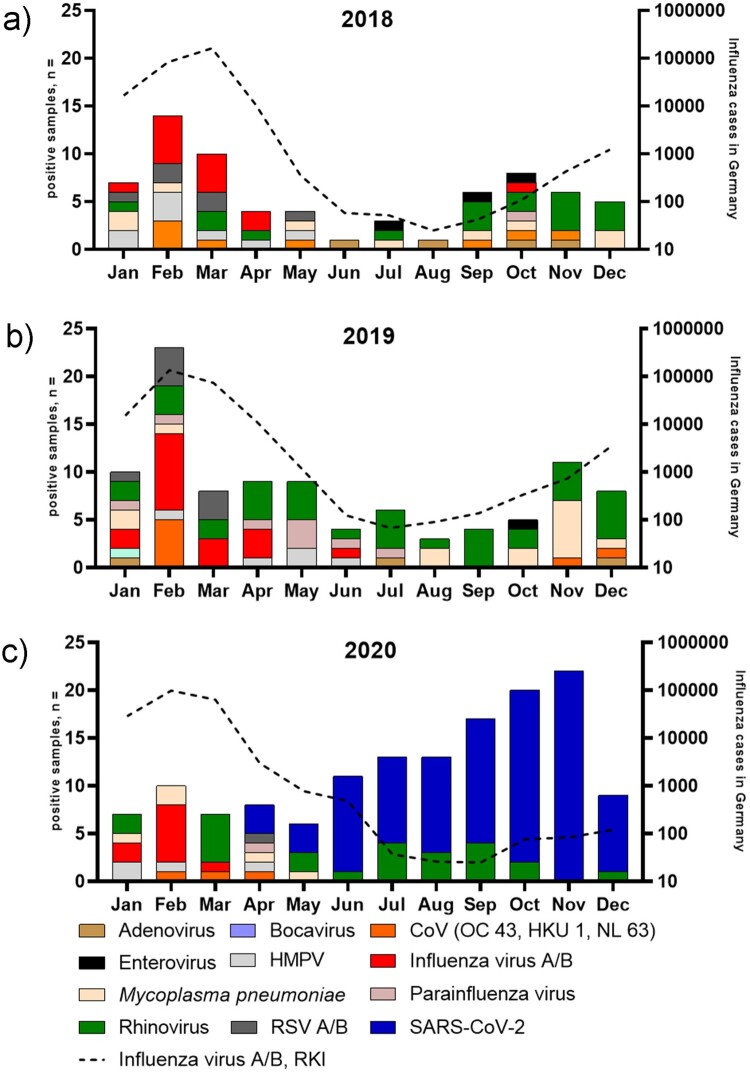
Histogram showing the total number of patients tested positive for a respiratory pathogen (left y-axis) using multiplex real-time RT-PCR per month in 2018 (Panel A), 2019 (Panel B), and 2020 [Panel C, including severe acute respiratory syndrome coronavirus 2 (SARS-CoV-2)]. Black broken line (Influenza virus A/B, RKI) denotes influenza case notifications submitted to the Robert-Koch-Institute, Germany (right y-axis, in log scale). Influenza A/B, RKI numbers were extracted from SurvStat software on 10th March 2021.

Here we have shown a pronounced shift in the pattern of acute respiratory tract pathogens in CAP patients with a replacement of common respiratory tract pathogens by SARS-CoV-2 in 2020. This is in contrast to previous seasons when, using the same molecular methods, picornaviruses, influenza virus and *Mycoplasma pneumoniae* dominated in CAP patients. The dominant role of influenza virus and rhinovirus in CAP patients is well established, whereas RSV, HMPV and parainfluenza virus seem to play a less prominent role [7].

Our data match very well with surveillance data showing the abrupt decline of influenza activity shortly after the appearance of SARS-CoV-2 and the initiation of NPI not only in Germany in early 2020 [8]. In addition, influenza activity remained very low in Europe until 31st December 2020 [9]. Of note, retrospective analysis of surveillance samples and samples from CAP patients did not detect unrecognized COVID-19 cases in Germany in December 2019 [5,10]. The strengths of our study are the complete and prospective coverage of three consecutive years using the same comprehensive molecular techniques. We used well-defined inclusion criteria, which remained unchanged throughout the complete study time, and we did not observe a drop in recruiting CAP patients during the COVID-19 pandemic. Of note, the possible effect of NPI has been shown previously [3]. Interestingly, rhinovirus seem to be less efficiently affected by these measures raising the question of how and why – Sullivan et al. discuss the possibility of rhinoviruses taking over the ecological niche that was previously occupied by RSV and influenza virus [4]. Leung et al. offer another plausible explanation in showing that rhinoviruses are not efficiently stopped by facemasks [11]. Moreover, rhinoviruses are also transmitted by direct contact, which may have been less effectively reduced by NPI compared to wearing facemasks [12]. Beyond the effect of NPI viral interference, influenza vaccination coverage (which has been very high in Germany in the winter season 2020/21), and globally reduced air travel have been discussed and might have contributed to the reduction of influenza cases in our cohort [4]. Cautiously, with the absence of exposure to influenza virus during the past winter, one might wonder if this will lead to more severe influenza seasons in the time to come [13].

We observed viral co-detections exclusively with rhinovirus in 7% of the SARS-CoV-2 positive patients. This further supports the notion that rhinoviruses are not efficiently withheld by facemasks or transmission occurred by direct route. Our findings are in contrast to Calcagno et al. who did not report viral co-detections among SARS-CoV2- patients and warrants further study [14].

As a limitation, we did not include data from conventional bacteriological analysis to determine the presence of bacterial (co)-infections. Thus, we did not consider the role of *Streptococcus pneumoniae* and *Haemophilus influenzae*, which constitute the leading organisms in CAP, in our study. Lower respiratory tract samples were not available to us, which might have led to an underestimation of the proportion of viral infections [7].

In conclusion, we show a marked shift in the prevalence of respiratory viral pathogens in CAP patients. Our data support the efficiency of NPI in stopping the spread of respiratory pathogens with the exception of rhinoviruses. The consequences regarding pathogen virulence and alteration of seasonality for a time after these measures remains uncertain. It has to be anticipated that waning population immunity might lead to a resurgence of respiratory cases as already evidenced by increasing and interseasonal RSV cases in the USA [15].
